# Antiplatelet therapy and the effects of B vitamins in patients with previous stroke or transient ischaemic attack: a post-hoc subanalysis of VITATOPS, a randomised, placebo-controlled trial

**DOI:** 10.1016/S1474-4422(12)70091-1

**Published:** 2012-06

**Authors:** Graeme J Hankey, John W Eikelboom, Qilong Yi, Kennedy R Lees, Christopher Chen, Denis Xavier, Jose C Navarro, Udaya K Ranawaka, Wasim Uddin, Stefano Ricci, John Gommans, Reinhold Schmidt, for the VITATOPS trial study group

**Affiliations:** a Department of Neurology, Royal Perth Hospital, Perth, WA, Australia; b Department of Medicine, McMaster University, Hamilton, ON, Canada; c National Epidemiology and Surveillance, Canadian Blood Services, Ottawa, ON, Canada; d Institute of Cardiovascular and Medical Sciences, University of Glasgow, Glasgow, UK; e Department of Pharmacology, National University of Singapore, Singapore; f Department of Pharmacology and Clinical Trials, St John's Medical College and Research Institute, Bangalore, India; g Jose R Reyes Memorial Medical Center, Manila, Philippines; h Faculty of Medicine, University of Kelaniya, Ragama, Sri Lanka; i Department of Medicine, POF Hospital, Wah Cantt, Pakistan; j UO Operativa di Neurologia, Azienda Sanitaria Locale 1 dell'Umbria, Italy; k Department of Medicine, Hawke's Bay Hospital, Hastings, New Zealand; l Department of Neurology, Medical University of Graz, Graz, Austria

## Abstract

**Background:**

Previous studies have suggested that any benefits of folic acid-based therapy to lower serum homocysteine in prevention of cardiovascular events might be offset by concomitant use of antiplatelet therapy. We aimed to establish whether there is an interaction between antiplatelet therapy and the effects of folic acid-based homocysteine-lowering therapy on major vascular events in patients with stroke or transient ischaemic attack enrolled in the vitamins to prevent stroke (VITATOPS) trial.

**Methods:**

In the VITATOPS trial, 8164 patients with recent stroke or transient ischaemic attack were randomly allocated to double-blind treatment with one tablet daily of placebo or B vitamins (2 mg folic acid, 25 mg vitamin B_6_, and 500 μg vitamin B_12_) and followed up for a median 3·4 years (IQR 2·0–5·5) for the primary composite outcome of stroke, myocardial infarction, or death from vascular causes. In our post-hoc analysis of the interaction between antiplatelet therapy and the effects of treatment with B vitamins on the primary outcome, we used Cox proportional hazards regression before and after adjusting for imbalances in baseline prognostic factors in participants who were and were not taking antiplatelet drugs at baseline and in participants assigned to receive B vitamins or placebo. We also assessed the interaction in different subgroups of patients and different secondary outcomes. The VITATOPS trial is registered with ClinicalTrials.gov, number NCT00097669, and Current Controlled Trials, number ISRCTN74743444.

**Findings:**

At baseline, 6609 patients were taking antiplatelet therapy and 1463 were not. Patients not receiving antiplatelet therapy were more likely to be younger, east Asian, and disabled, to have a haemorrhagic stroke or cardioembolic ischaemic stroke, and to have a history of hypertension or atrial fibrillation. They were less likely to be smokers and to have a history of peripheral artery disease, hypercholesterolaemia, diabetes, ischaemic heart disease, and a revascularisation procedure. Of the participants taking antiplatelet drugs at baseline, B vitamins had no significant effect on the primary outcome (488 patients in the B-vitamins group [15%] *vs* 519 in the placebo group [16%]; hazard ratio [HR] 0·94, 95% CI 0·83–1·07). By contrast, of the participants not taking antiplatelet drugs at baseline, B vitamins had a significant effect on the primary outcome (123 in the B-vitamins group [17%] *vs* 153 in the placebo group [21%]; HR 0·76, 0·60–0·96). The interaction between antiplatelet therapy and the effect of B vitamins on the primary outcome was significant after adjusting for imbalance in the baseline variables (adjusted p for interaction=0·0204).

**Interpretation:**

Our findings support the hypothesis that antiplatelet therapy modifies the potential benefits of lowering homocysteine with B-vitamin supplementation in the secondary prevention of major vascular events. If validated, B vitamins might have a role in the prevention of ischaemic events in high-risk individuals with an allergy, intolerance, or lack of indication for antiplatelet therapy.

**Funding:**

Australia National Health and Medical Research Council, UK Medical Research Council, Singapore Biomedical Research Council, and Singapore National Medical Research Council.

## Introduction

Observational studies show a strong, positive, and dose-related association between serum concentrations of homocysteine and the risk of stroke, which is independent of other vascular risk factors and biologically plausible.^1^, ^2^ Homocysteine can be lowered by a mean of 25% (95% CI 23–28) with folic acid supplementation.^3^ A meta-analysis of eight randomised, placebo-controlled trials of folic acid supplementation in 37 485 patients^4^ showed that, despite yielding an average 25% reduction in homocysteine, folic acid had no significant effect on the rate of first stroke (rate ratio 0·96, 95% CI 0·87–1·06) over a median follow-up of 5 years. However, the role of homocysteine-lowering in stroke prevention might be complex.^5^ A meta-analysis of 237 genetic epidemiological studies,^6^ in which homocysteine and the presence of the methylene tetrahydrofolate reductase C677T polymorphism in 60 000 individuals were correlated with 20 885 subsequent stroke events, suggested that established or increasing dietary folate intake in the countries where the trials were undertaken might have modified the effect of lowering homocysteine on risk of stroke.^6^

Antiplatelet therapy might also modify the effect of lowering homocysteine on the risk of stroke and ischaemic heart disease events.^7^, ^8^, ^9^ An exploratory analysis of trials of lowering homocysteine^7^ suggested an interaction between antiplatelet therapy and the effect of lowering homocysteine on risk of ischaemic heart disease events: in the five trials with the lowest prevalence of antiplatelet therapy (mean 60%, usually aspirin), the relative risk was 0·93 (95% CI 0·84–1·05) and in the five trials with the highest prevalence (mean 91%) the relative risk was 1·09 (1·00–1·19), p for interaction=0·037. In another analysis of trials of the effects of lowering homocysteine on the risk of stroke events,^8^ the effect was greater in the four trials that enrolled patients with renal disease and oesophageal dysplasia (who were not likely to be taking antiplatelet therapy) compared with the trials that enrolled patients with previous vascular disease. The Heart Outcomes Prevention Evaluation 2 (HOPE 2) trial^9^ subsequently reported a non-significant trend towards a greater effect of folic acid-based vitamin B supplementation, compared with placebo, in reducing stroke in patients with known cardiovascular disease who were not taking antiplatelet therapy at enrolment compared with patients who were (p for interaction=0·25). The biological plausibility of these findings is supported by the recognised potential for antiplatelet therapy to modify any antithrombotic or other antiatherogenic effects of lowering homocysteine.^10^, ^11^, ^12^, ^13^

These analyses prompted us to undertake a post-hoc subanalysis of the vitamins to prevent stroke (VITATOPS) trial. We aimed to explore the hypothesis that there is an interaction between antiplatelet therapy and the effect of folic acid-based vitamin B supplementation on major vascular events in the VITATOPS trial population of patients with previous stroke or transient ischaemic attack.^14^

## Methods

### Participants

The methods and primary results of the VITATOPS trial have been reported.^14^ Briefly, the VITATOPS trial was a randomised, double-blind, parallel, placebo-controlled trial in which 8164 patients were recruited from 123 centres in 20 countries of four continents, and randomly assigned to take one tablet daily of placebo or B vitamins (2 mg folic acid, 25 mg vitamin B_6_, 500 μg vitamin B_12_). Patients were eligible for inclusion if they had a stroke (ischaemic or haemorrhagic) or transient ischaemic attack (eye or brain) within the past 7 months.

Patients were excluded if they were taking folic acid, vitamin B_6_, vitamin B_12_, or a folate antagonist (eg, methotrexate), if they were pregnant or were women of childbearing potential, or if they had a restricted life expectancy (eg, because of ill health).

At enrolment, participants were asked if they were taking antiplatelet drugs (eg, aspirin, clopidogrel, dipyridamole). The trial received ethical approval from national (India, New Zealand, and the UK) and local research ethics committees and all patients provided written informed consent before enrolment.

### Procedures

Patients were randomly assigned (1:1) to receive either B vitamins or matching placebo by means of a central 24 h telephone service or an interactive website in which random permuted blocks were stratified by hospital. Treatment groups were masked from patients and investigators. Randomisation was not stratified in accordance with the presence or absence of antiplatelet therapy. The primary outcome was the composite of any stroke, myocardial infarction, or death from vascular causes.

### Statistical analysis

We tabulated baseline characteristics and laboratory data in accordance with the presence or absence of antiplatelet therapy at baseline and in accordance with the assigned treatment groups, and expressed them as proportions for categorical variables and means for continuous variables. We compared categorical variables in each group with the χ^2^ test, and continuous variables with the *t* test. We calculated event rates as the number of events during the follow-up period divided by the total number of patients that entered randomisation.

We constructed Kaplan-Meier curves to show the cumulative effects of B vitamins compared with placebo on the primary outcome in participants who were and were not taking antiplatelet therapy at baseline.

We assessed the interaction between antiplatelet therapy and the effects of treatment with B vitamins on the primary outcome by means of Cox proportional hazards regression before and after adjusting for imbalances in important baseline prognostic factors in participants who were and were not taking antiplatelet drugs at baseline, and in participants assigned B vitamins or placebo. We also assessed the consistency of the interaction effect in different subgroups of patients, and in different secondary outcome events including ischaemic stroke, haemorrhagic stroke, myocardial infarction, and death from vascular causes.

We adjusted for certain variables in our models: age, sex, ethnic origin, pathological and causal subtypes of stroke and transient ischaemic attack, stroke severity as measured by the Oxford handicap score, smoking, treated and untreated hypercholesterolaemia, and history of stroke, myocardial infarction, ischaemic heart disease, peripheral arterial disease, atrial fibrillation, and diabetes.

We compared the mean serum concentrations of homocysteine and vitamin B_12_ and mean red-cell concentration of folate, which were measured at both baseline and follow-up in the same individual, with a paired *t* test. We calculated the difference between baseline and follow-up measures, and tested the interaction effect between antiplatelet use at baseline and treatment allocation with a linear regression model.

We used two-sided significance tests throughout and we deemed a two-sided p value of less than 0·05 to be significant. The VITATOPS trial is registered with ClinicalTrials.gov, number NCT00097669, and Current Controlled Trials, number ISRCTN74743444.

### Role of the funding source

The sponsors of the study had no role in study design, data collection, data analysis, data interpretation, the writing of the report, or in the decision to submit the paper for publication. The corresponding author had full access to all the data in the study and had final responsibility for the decision to submit for publication.

## Results

At baseline, 6609 patients (81%) were in receipt of antiplatelet therapy, 1463 (18%) were not, and in 92 (1%) antiplatelet therapy status was not known. The composite primary outcome of stroke, myocardial infarction, or death from vascular causes was recorded in 616 patients (15%) assigned to receive B vitamins and 678 (17%) assigned to receive placebo (risk ratio 0·91, 95% CI 0·82 to 1·00, p=0·05; absolute risk reduction 1·56%, 95% CI −0·01 to 3·16).^14^

Compared with patients receiving antiplatelet therapy, patients who were not receiving antiplatelet therapy at baseline were more likely to be younger, east Asian, and disabled, to have a haemorrhagic stroke or cardioembolic ischaemic stroke, and to have a history of hypertension or atrial fibrillation (table 1). They were less likely to be smokers and to have a history of peripheral vascular disease, hypercholesterolaemia, diabetes, ischaemic heart disease, and a revascularisation procedure. Of patients who were or were not receiving antiplatelet therapy at baseline, baseline characteristics were evenly distributed between patients assigned to receive either B vitamins or placebo (table 2).

**Table 1:** Baseline characteristics

			**Antiplatelet treatment (N=6609)**	**No antiplatelet treatment (N=1463)**	**p value**
Age (years)			62·9 (12·3)	61·1 (13·2)	<0·0001
Men			4227 (64·0%)	922 (63·0%)	0·4910
Women			2380 (36·0%)	541 (37·0%)	..
Ethnic origin					
	White		2755 (43·2%)	511 (36·3%)	<0·0001
	East Asian		1455 (22·8%)	445 (31·6%)	..
	South Asian		1733 (27·2%)	316 (22·4%)	..
	Other		435 (6·8%)	136 (9·7%)	..
Oxfordshire classification of stroke subtype					
	Total anterior circulation syndrome		132 (2·0%)	60 (4·1%)	<0·0001
	Partial anterior circulation syndrome		3512 (53·7%)	780 (53·9%)	..
	Lacunar syndrome		2516 (38·5%)	511 (35·3%)	..
	Posterior circulation syndrome		382 (5·8%)	95 (6·6%)	..
Pathological subtype of stroke					
	Transient ischaemic attack		1250 (18·9%)	146 (10·0%)	<0·0001
	Ischaemic stroke		5117 (77·5%)	574 (39·3%)	..
	Intracerebral haemorrhage		82 (1·2%)	654 (44·8%)	..
	Subarachnoid haemorrhage		10 (0·2%)	56 (3·8%)	..
	Retinal infarction		16 (0·2%)	2 (0·1%)	..
	Unknown or uncertain pathology		126 (1·9%)	29 (2·0%)	..
Causal subtype of stroke					
	Large artery disease		2788 (42·5%)	227 (15·6%)	<0·0001
	Small artery disease		2555 (38·9%)	203 (14·0%)	..
	Embolism from the heart		190 (2·9%)	209 (14·4%)	..
	Uncertain or unknown		911 (13·9%)	97 (6·7%)	..
	Haemorrhagic event		118 (1·8%)	718 (49·4%)	..
Oxford handicap score					
	2 or less (independent)		5136 (79·0%)	902 (63·2%)	<0·0001
	3 or greater (dependent)		1366 (21·0%)	525 (36·8%)	..
Medical history					
	Stroke		1041 (15·8%)	233 (16·1%)	0·7732
	Myocardial infarction		501 (7·6%)	95 (6·6%)	0·1797
	Peripheral arterial disease		321 (4·9%)	44 (3·0%)	0·0024
	Revascularisation procedure of brain, heart, or limbs		482 (7·3%)	82 (5·6%)	0·0219
	Hypertension*		4634 (70·3%)	1081 (74·7%)	0·0009
		Treated hypertension event	3631 (55·3%)	783 (54·2%)	0·4516
	Smoking		3337 (50·7%)	671 (46·4%)	0·0033
		Present smoker or at time of event	1615 (24·6%)	288 (19·9%)	0·0001
	Hypercholesterolaemia†		2315 (35·2%)	330 (22·9%)	<0·0001
		Treated hypercholesterolaemia event	2001 (30·6%)	266 (18·6%)	<0·0001
	Diabetes mellitus		1641 (24·9%)	254 (17·5%)	<0·0001
	Atrial fibrillation		333 (5·1%)	313 (21·6%)	<0·0001
	Ischaemic heart disease		1126 (17·6%)	197 (14·1%)	0·0014
	History of depression		451 (7·6%)	92 (7·0%)	0·4947
	Alcohol intake (standard drinks [10 g alcohol] per day)		0·8 (2·5)	0·9 (2·5)	0·1888

Data are mean (SD) or n (%).

* History of hypertension or treated hypertension at randomisation.

† History of hypercholesterolaemia (>6·5 mmol/L) or treated hypercholesterolaemia at randomisation.

**Table 2:** Baseline characteristics by treatment allocation

			**Antiplatelet treatment (N=6609)**		**No antiplatelet treatment (N=1463)**	
			Placebo group (n=3303)	B-vitamins group (n=3306)	Placebo group (n=729)	B-vitamins group (n=734)
Age (years)			63·0 (12·2)	62·8 (12·4)	61·3 (13·0)	61·0 (13·3)
Men			2097 (63·5%)	2130 (64·4%)	475 (65·2%)	447 (60·9%)
Women			1205 (36·5%)	1175 (35·6%)	254 (34·8%)	287 (39·1%)
Ethnic origin						
	White		1378 (43·3%)	1377 (43·1%)	257 (36·5%)	254 (36·1%)
	East Asian		732 (23·0%)	723 (22·6%)	217 (30·8%)	228 (32·4%)
	South Asian		857 (26·9%)	876 (27·4%)	159 (22·6%)	157 (22·3%)
	Other		215 (6·8%)	220 (6·9%)	71 (10·1%)	65 (9·2%)
Oxfordshire classification of stroke subtype						
	Total anterior circulation syndrome		71 (2·2%)	61 (1·9%)	31 (4·3%)	29 (4·0%)
	Partial anterior circulation syndrome		1758 (53·8%)	1754 (53·6%)	390 (54·1%)	390 (53·8%)
	Lacunar syndrome		1256 (38·4%)	1260 (38·5%)	253 (35·1%)	258 (35·6%)
	Posterior circulation syndrome		184 (5·6%)	198 (6·0%)	47 (6·5%)	48 (6·6%)
Pathological subtype of stroke						
	Transient ischaemic attack		634 (19·2%)	616 (18·7%)	80 (11·0%)	66 (9·0%)
	Ischaemic stroke		2560 (77·6%)	2557 (77·4%)	278 (38·2%)	296 (40·4%)
	Intracerebral haemorrhage		37 (1·1%)	45 (1·4%)	317 (43·5%)	337 (46·0%)
	Subarachnoid haemorrhage		4 (0·1%)	6 (0·2%)	30 (4·1%)	26 (3·5%)
	Retinal infarction		9 (0·3%)	7 (0·2%)	2 (0·3%)	0 (0%)
	Unknown or uncertain pathology		55 (1·7%)	71 (2·2%)	21 (2·9%)	8 (1·1%)
Causal subtype of stroke						
	Large artery disease		1405 (42·9%)	1383 (42·1%)	118 (16·3%)	109 (15·0%)
	Small artery disease		1281 (39·1%)	1274 (38·8%)	104 (14·3%)	99 (13·6%)
	Embolism from the heart		88 (2·7%)	102 (3·1%)	97 (13·4%)	112 (15·4%)
	Uncertain or unknown		453 (13·8%)	458 (13·9%)	54 (7·4%)	43 (5·9%)
	Haemorrhagic event		50 (1·5%)	68 (2·1%)	353 (48·6%)	365 (50·1%)
Oxford handicap score						
	2 or less (independent)		2556 (78·7%)	2580 (79·2%)	461 (64·8%)	441 (61·6%)
	3 or greater (dependent)		690 (21·3%)	676 (20·8%)	250 (35·2%)	275 (38·4%)
Medical history						
	Stroke		528 (16·0%)	513 (15·6%)	126 (17·5%)	107 (14·8%)
	Myocardial infarction		255 (7·8%)	246 (7·5%)	45 (6·3%)	50 (6·9%)
	Peripheral arterial disease		163 (5·0%)	158 (4·8%)	25 (3·5%)	19 (2·6%)
	Revascularisation procedure of brain, heart, or limbs		248 (7·5%)	234 (7·1%)	44 (6·0%)	38 (5·2%)
	Hypertension*		2330 (70·7%)	2304 (69·9%)	534 (74·0%)	547 (75·4%)
		Treated hypertension event	1812 (55·2%)	1819 (55·4%)	390 (54·2%)	393 (54·2%)
	Smoking		1669 (50·7%)	1668 (50·6%)	332 (45·9%)	339 (46·9%)
		Present smoker or at time of event	806 (24·6%)	809 (24·6%)	138 (19·1%)	150 (20·7%)
	Hypercholesterolaemia†		1157 (35·1%)	1158 (35·2%)	161 (22·4%)	169 (23·4%)
		Treated hypercholesterolaemia event	987 (30·2%)	1014 (31·0%)	135 (19·0%)	131 (18·2%)
	Diabetes mellitus		823 (25·0%)	818 (24·8%)	121 (16·7%)	133 (18·3%)
	Atrial fibrillation		165 (5·0%)	168 (5·1%)	152 (21·1%)	161 (22·2%)
	Ischaemic heart disease		573 (18·0%)	553 (17·3%)	96 (13·7%)	101 (14·5%)
	History of depression		218 (7·3%)	233 (7·8%)	52 (8·0%)	40 (6·1%)
	Alcohol intake (standard drinks [10 g alcohol] per day)		0·9 (2·7)	0·8 (2·2)	0·8 (2·2)	1·0 (2·8)

Data are mean (SD) or n (%).

* History of hypertension or treated hypertension at randomisation.

† History of hypercholesterolaemia (>6·5 mmol/L) or treated hypercholesterolaemia at randomisation.

Baseline antiplatelet therapy was an independent significant predictor of a lower rate of subsequent stroke, myocardial infarction, or death from vascular causes in all patients who entered randomisation (hazard ratio [HR] 0·66, 95% CI 0·55–0·81).

Of the 6609 participants in receipt of antiplatelet drugs at baseline, the primary outcome was recorded in roughly 15% of participants assigned to receive B vitamins or placebo (table 3). By contrast, of the 1463 participants who were not in receipt of antiplatelet drugs at baseline, the primary outcome was recorded in slightly more participants in the placebo group (table 3). After adjusting for the effects of imbalance in baseline variables, the HR for the primary outcome for patients assigned B vitamins versus placebo was greater for participants taking antiplatelet therapy than for those who were not (table 3).

**Table 3:** Interaction between B-vitamin supplementation and antiplatelet therapy at baseline on each major vascular outcome

	**B-vitamins group**		**Placebo group**		**Hazard ratio (95% CI)**	**p for interaction**	Adjusted hazard ratio (95% CI)*	Adjusted p for interaction*
	Total	n (%)	Total	n (%)				
**Stroke, myocardial infarction, or vascular death**								
Antiplatelet use	3306	488 (14·8%)	3303	519 (15·7%)	0·94 (0·83–1·07)	0·0980	0·98 (0·86–1·11)	0·0204
No antiplatelet use	734	123 (16·8%)	729	153 (21·0%)	0·76 (0·60–0·96)		0·71 (0·55–0·90)	
**Stroke**								
Antiplatelet use	3306	293 (8·9%)	3303	297 (9·0%)	0·99 (0·84–1·17)	0·0452	1·03 (0·87–1·22)	0·0134
No antiplatelet use	734	65 (8·9%)	729	89 (12·2%)	0·69 (0·50–0·95)		0·65 (0·46–0·91)	
**Vascular death**								
Antiplatelet use	3306	254 (7·7%)	3303	278 (8·4%)	0·92 (0·78–1·10)	0·0838	0·96 (0·81–1·16)	0·0225
No antiplatelet use	734	70 (9·5%)	729	97 (13·3%)	0·68 (0·50–0·93)		0·63 (0·46–0·88)	
**Myocardial infarction**								
Antiplatelet use	3306	98 (3·0%)	3303	95 (2·9%)	1·04 (0·78–1·37)	0·6630	0·97 (0·72–1·31)	0·9588
No antiplatelet use	734	18 (2·5%)	729	19 (2·6%)	0·90 (0·47–1·72)		0·89 (0·45–1·79)	
**Stroke or vascular death**								
Antiplatelet use	3306	453 (13·7%)	3303	476 (14·4%)	0·96 (0·84–1·09)	0·0553	0·99 (0·87–1·14)	0·0072
No antiplatelet use	734	113 (15·4%)	729	145 (19·9%)	0·74 (0·57–0·94)		0·68 (0·52–0·88)	

* Adjusted for age, sex, ethnic origin, history of stroke, myocardial infarction, hypertension, ischaemic heart disease, peripheral arterial disease, diabetes, cholesterol, smoking status, Oxford handicap score, pathology, and cause of stroke and transient ischaemic attack.

The figure shows Kaplan-Meier curves of the cumulative probability of the primary outcome event in patients who were and were not taking antiplatelet at the time of randomisation into the VITATOPS trial. In table 3 we also show the results for the individual components of the primary outcome. The overall results for the primary outcome were consistent for stroke and for vascular death, but not for myocardial infarction.

**Figure:**
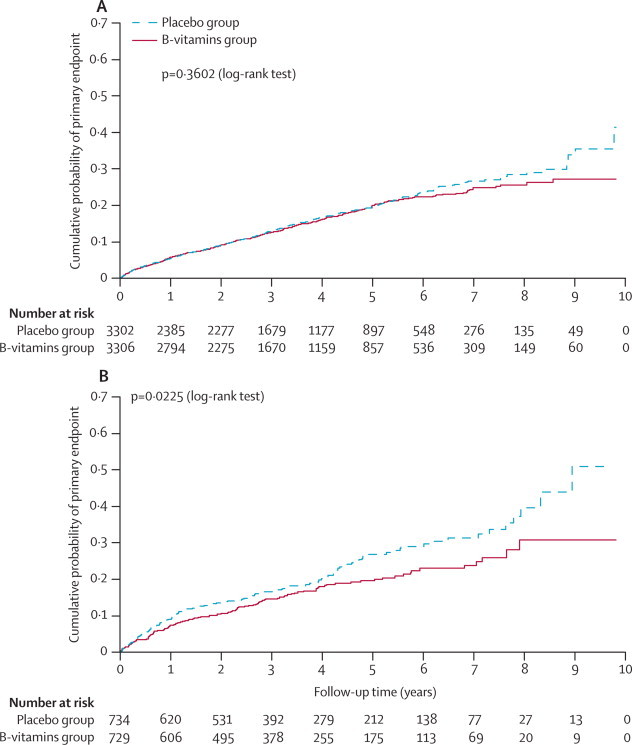
Kaplan-Meier curves of the cumulative probability of the primary outcome event Cumulative probability of stroke, myocardial infarction, or death from vascular causes in patients with previous stroke or transient ischaemic attack who were (A) or were not (B) in receipt of antiplatelet therapy at the time of randomisation into the VITATOPS trial.

In table 4 we show a significant interaction between antiplatelet use at baseline and the effect of B vitamins on recurrent ischaemic stroke after adjustment for baseline factors. The trend was similar, but not significant, for recurrent haemorrhagic stroke.

**Table 4:** Interaction between B-vitamin supplementation and antiplatelet therapy at baseline on recurrent stroke subtypes

		**B-vitamins group**		**Placebo group**		**Hazard ratio (95% CI)**	**p for interaction**	Adjusted hazard ratio (95% CI)*	Adjusted p for interaction*
		Total	n (%)	Total	n (%)				
**All patients**†									
Recurrent stroke (ischaemic; first ever or recurrent)									
	Antiplatelet use	3306	212 (6·4%)	3303	187 (5·7%)	1·14 (0·94–1·39)	0·1154	1·16 (0·94–1·43)	0·0392
	No antiplatelet use	734	36 (4·9%)	729	44 (6·0%)	0·78 (0·50–1·21)		0·69 (0·44–1·11)	
Recurrent stroke (haemorrhagic; first ever or recurrent)									
	Antiplatelet use	3306	26 (0·8%)	3303	24 (0·7%)	1·09 (0·63–1·90)	0·0866	1·10 (0·61–1·97)	0·0757
	No antiplatelet use	734	15 (2·0%)	729	27 (3·7%)	0·52 (0·28–0·99)		0·48 (0·24–0·94)	
**Patients with only non-haemorrhagic stroke or transient ischaemic attack**‡									
Recurrent stroke (ischaemic; first ever or recurrent)									
	Antiplatelet use	3255	211 (6·5%)	3262	187 (5·7%)	1·14 (0·93–1·38)	0·3208	1·16 (0·94–1·43)	0·1193
	No antiplatelet use	371	29 (7·8%)	382	33 (8·6%)	0·88 (0·53–1·44)		0·75 (0·44–1·29)	
Recurrent stroke (haemorrhagic; first ever or recurrent)									
	Antiplatelet use	3255	25 (0·7%)	3262	24 (0·7%)	1·05 (0·60–1·84)	0·5815	1·06 (0·59–1·93)	0·6016
	No antiplatelet use	371	7 (1·9%)	382	9 (2·4%)	0·76 (0·28–2·05)		0·69 (0·233–2·04)	

* Adjusted for age, sex, ethnic origin, history of stroke, myocardial infarction, hypertension, ischaemic heart disease, peripheral arterial disease, diabetes, cholesterol, smoking status, Oxford handicap score, pathology, and cause of stroke and transient ischaemic attack.

† Qualifying event was ischaemic or haemorrhagic stroke or transient ischaemic attack.

‡ Qualifying event was only ischaemic stroke or transient ischaemic attack.

In table 5 we show that of all the listed subgroups, with the exception of participants with cardioembolic ischaemic stroke, the HR for the effect of B vitamins compared with placebo on the primary outcome was lower in patients who were not in receipt of antiplatelet therapy at baseline than in patients who were, but many of the comparisons were not statistically significant.

**Table 5:** Interaction between B-vitamin supplementation and antiplatelet therapy at baseline on the primary outcome stratified by baseline characteristics

	**B-vitamins group**		**Placebo group**		**Hazard ratio (95% CI)**	**p for interaction**	Adjusted p for interaction*
	Total	n (%)	Total	n (%)			
**Age <60 years**							
Antiplatelet use	1237	122 (9·9%)	1208	135 (11·2%)	0·89 (0·70–1·13)	0·4957	0·3521
No antiplatelet use	325	34 (10·5%)	319	43 (13·5%)	0·75 (0·48–1·17)		
**Age between 60–69 years**							
Antiplatelet use	960	123 (12·8%)	995	139 (14·0%)	0·90 (0·72–1·17)	0·6991	0·4442
No antiplatelet use	206	43 (16·5%)	200	38 (19·0%)	0·83 (0·52–1·32)		
**Age >69 years**							
Antiplatelet use	1109	243 (21·9%)	1100	245 (22·3%)	1·00 (0·84–1·20)	0·1018	0·0379
No antiplatelet use	203	55 (27·1%)	210	72 (34·3%)	0·73 (0·51–1·03)		
**Transient ischaemic attack**							
Antiplatelet use	616	63 (10·2%)	634	83 (13·1%)	0·79 (0·57–1·09)	0·2669	0·3513
No antiplatelet use	66	6 (9·1%)	80	17 (21·3%)	0·48 (0·19–1·22)		
**Ischaemic stroke**							
Antiplatelet use	2557	405 (15·8%)	2560	416 (16·3%)	0·98 (0·85–1·12)	0·5673	0·2114
No antiplatelet use	296	75 (25·3%)	278	73 (26·3%)	0·90 (0·65–1·24)		
**Non-haemorrhagic stroke or transient ischaemic attack**							
Antiplatelet use	3255	481 (14·8%)	3262	512 (15·7%)	0·94 (0·83–1·07)	0·5907	0·1159
No antiplatelet use	371	82 (22·10%)	382	93 (24·4%)	0·87 (0·65–1·17)		
**Intracerebral haemorrhage**							
Antiplatelet use	45	6 (13·3%)	37	7 (18·9%)	0·72 (0·24–2·14)	0·5842	0·8060
No antiplatelet use	337	39 (11·6%)	317	57 (18·0%)	0·58 (0·39–0·88)		
**Subarachnoid haemorrhage**							
Antiplatelet use	6	1 (16·7%)	4	0 (0·0%)	..	..	..
No antiplatelet use	26	2 (7·7%)	30	3 (10·0%)	0·80 (0·13–4·80)		
**Large artery disease**							
Antiplatelet use	1383	255 (18·4%)	1405	232 (16·5%)	1·13 (0·95–1·35)	0·2104	0·0438
No antiplatelet use	109	24 (22·0%)	118	31 (26·3%)	0·81 (0·47–1·37)		
**Small artery disease**							
Antiplatelet use	1274	167 (13·1%)	1281	206 (16·1%)	0·80 (0·65–0·98)	0·5589	0·8683
No antiplatelet use	99	23 (23·3%)	104	33 (31·7%)	0·67 (0·39–1·14)		
**Embolism from the heart**							
Antiplatelet use	102	22 (21·6%)	88	27 (30·7%)	0·64 (0·37–1·13)	0·1576	0·8186
No antiplatelet use	112	27 (24·1%)	97	21 (21·7%)	1·14 (0·64–2·01)		
**Smoking**							
Antiplatelet use	1668	279 (16·7%)	1669	275 (16·5%)	1·03 (0·87–1·22)	0·0633	0·0553
No antiplatelet use	339	61 (18·0%)	332	81 (24·4%)	0·73 (0·52–1·02)		
**No smoking**							
Antiplatelet use	1626	206 (12·7%)	1623	242 (14·9%)	0·84 (0·70–1·01)	0·7663	0·1333
No antiplatelet use	384	61 (15·9%)	391	71 (18·2%)	0·80 (0·57–1·13)		
**Diabetes**							
Antiplatelet use	818	147 (18·0%)	823	153 (18·6%)	1·00 (0·79–1·25)	0·0555	0·0205
No antiplatelet use	133	32 (24·1%)	121	43 (35·5%)	0·61 (0·39–0·97)		
**No diabetes**							
Antiplatelet use	2480	339 (13·7%)	2471	364 (14·7%)	0·93 (0·80–1·08)	0·3179	0·1520
No antiplatelet use	593	89 (15·0%)	602	109 (18·1%)	0·80 (0·60–1·06)		
**High cholesterol (≥6·5 mmol/L)**							
Antiplatelet use	1158	170 (14·7%)	1157	184 (15·9%)	0·92 (0·75–1·14)	0·3346	0·2473
No antiplatelet use	169	29 (17·2%)	161	36 (22·4%)	0·72 (0·44–1·18)		
**Normal cholesterol (<6·5 mmol/L)**							
Antiplatelet use	1496	214 (14·3%)	1463	209 (14·3%)	1·03 (0·85–1·25)	0·0265	0·0069
No antiplatelet use	354	51 (14·4%)	377	75 (19·9%)	0·66 (0·46–0·94)		
**Treated high cholesterol**							
Antiplatelet use	1014	144 (14·2%)	987	160 (16·2%)	0·88 (0·70–1·10)	0·1825	0·2762
No antiplatelet use	131	22 (16·8%)	135	25 (25·2%)	0·60 (0·35–1·03)		
**Untreated high cholesterol**							
Antiplatelet use	2261	339 (15·0%)	2283	356 (15·6%)	0·97 (0·84–1·13)	0·2154	0·0234
No antiplatelet use	589	97 (16·5%)	577	114 (19·8%)	0·81 (0·62–1·07)		

* Adjusted for age, sex, ethnic origin, history of stroke, myocardial infarction, hypertension, ischaemic heart disease, peripheral arterial disease, diabetes, cholesterol, smoking status, Oxford handicap score, pathology, and cause of stroke and transient ischaemic attack.

In table 6 we show that supplementation with B vitamins significantly lowered total homocysteine and increased red cell folate concentration during follow-up in patients who were and were not in receipt of antiplatelet therapy at baseline. Supplementation with B vitamins also significantly increased serum vitamin B_12_ concentration during follow-up in patients in receipt of antiplatelet therapy at baseline, but the effect was not significant for patients not receiving antiplatelet therapy at baseline. The effects of supplementation with B vitamins on lowering total homocysteine and increasing red-cell folate and vitamin B_12_ concentration were not significantly different between patients who were and were not in receipt of antiplatelet therapy at baseline. The p for interaction between antiplatelet therapy at baseline and trial treatment was 0·2501 for total homocysteine, 0·8996 for red cell folate, and 0·6591 for vitamin B_12_.

**Table 6:** Homocysteine, red cell folate, and vitamin B_12_ concentrations at baseline and during follow-up

	**Antiplatelet treatment**			**No antiplatelet treatment**		
	Baseline	Follow-up	Difference (95% CI); p value*	Baseline	Follow-up	Difference (95% CI); p value*
**Homocysteine (μmol/L)**						
B-vitamins group	13·7 (6·6)	10·5 (4·4)	−3·18 (−2·66 to −3·70); p<0·0001	12·4 (4·3)	9·9 (2·6)	−2·46 (−1·46 to −3·46); p<0·0001
Placebo group	13·4 (4·9)	14·4 (5·8)	0·94 (0·40 to 1·47); p=0·0006	13·3 (5·8)	13·8 (5·1)	0·56 (−0·50 to 1·63); p=0·2937
**Red cell folate (nmol/L)**						
B-vitamins group	971·6 (464·6)	2297·9 (789·4)	1326·2 (1195·8 to 1456·6); p<0·0001	906·8 (432·7)	2090·9 (752·3)	1184·1 (951·1 to 1417·2); p<0·0001
Placebo group	867·0 (445·3)	1156·5 (686·0)	289·5 (186·9 to 392·1); p<0·0001	990·7 (515·9)	1112·9 (628·7)	122·2 (−109·2 to 353·5); p=0·2901
**Vitamin B_12_ (pmol/L)**						
B-vitamins group	312·3 (139·3)	367·5 (195·6)	55·1 (21·3 to 89·0); p=0·0016	368·6 (192·4)	396·9 (239·4)	28·4 (−67·0 to 123·7); p=0·5494
Placebo group	311·9 (127·5)	205·7 (132·2)	−106·2 (−79·7 to −132·8); p<0·0001	342·7 (127·5)	186·4 (90·7)	−156·3 (−109·8 to −202·8); p<0·0001

Data are mean (SD) unless otherwise stated.

* Comparison between baseline and during the follow-up was undertaken with a paired *t* test. Some of the follow-up measures were taken during follow-up (eg, at the regular follow-up assessments every 6 months) and some at the end of follow-up.

After excluding patients with a qualifying diagnosis of haemorrhagic stroke, the interaction between B vitamins and antiplatelet therapy was not significant (adjusted p=0·1159), but the adjusted HR for B vitamins versus placebo on the primary outcome in participants not in receipt of antiplatelet therapy at baseline was still lower (HR 0·75, 95% CI 0·54–1·03) than in participants who were in receipt of therapy (0·98, 0·86–1·12). We also did a matched paired analysis, and a similar pattern was evident.

## Discussion

The principal result of the VITATOPS trial was that daily administration of B vitamins to patients with recent stroke or transient ischaemic attack for a median of 3·4 years had no significant effect, compared with placebo, on the overall incidence of major vascular events. However, our post-hoc subanalysis supports hypotheses from previous independent trials of lowering total homocysteine on both ischaemic heart disease and stroke outcome events that antiplatelet therapy, which was taken by most patients, might have modified any favourable effect of folic acid supplementation on major vascular events (panel).^7^, ^9^

Panel **Research in context**

**Systematic review**

We searched PubMed with the terms “homocysteine”, “folic acid”, “vitamins”, “antiplatelet”, “aspirin”, “clopidogrel”, “dipyridamole”, “cilostazol”, “stroke”, “ischaemic heart disease”, “major vascular events”, “interaction”, “randomised trial”, and “clinical trial” for reports of an interaction between antiplatelet therapy and treatments that lower homocysteine in the prevention of stroke and other major vascular events. We searched for work published before March, 2012. The quality of evidence we required was a randomised, controlled trial or meta-analysis of such trials. We identified the Heart Outcomes Prevention Evaluation 2 trial^9^ and the meta-analysis of randomised trials of lowering total homocysteine on risk of ischaemic heart disease events^7^ as directly relevant, and a further meta-analysis^8^ as indirectly relevant.

**Interpretation**

The results of our exploratory analyses of the VITATOPS trial support previous hypotheses that antiplatelet therapy, which was taken by most patients, might modify any favourable effect of folic acid supplementation on major vascular events.^7^, ^9^ If our finding are validated in independent studies, B vitamins might have a role in the prevention of vascular events in high-risk individuals with an allergy, intolerance, or lack of indication for antiplatelet therapy, such as those with haemorrhagic stroke.

The VITATOPS trial had several strengths: systematic bias in treatment allocation was minimised by the randomisation process; observer bias in the assessment of vascular outcomes was minimised by the masking of treatment allocation from assessors, clinicians, and patients; and random error was reduced by the reasonably large number of outcome events. The strengths of our analysis are that it was based on a pre-existing hypothesis (that antiplatelet therapy might interact with the effect of B vitamins on vascular risk), the hypothesis is plausible, the interaction between B-vitamin supplementation and only one subgroup was assessed (antiplatelet use at baseline or not; table 3), the primary trial outcome was the main outcome studied, the distribution of important prognostic factors was reasonably, although not perfectly, balanced between treatment groups within each subgroup (table 2), the analysis was based on appropriate statistical tests of subgroup-treatment effect interaction, all subgroup analyses that were undertaken have been reported, and the results have been interpreted cautiously on the premise that subgroup analyses are intrinsically limited.^15^

Potential limitations are that, because this substudy was not a primary aim or prespecified analysis of the VITATOPS trial, the type of antiplatelet therapy taken (eg, aspirin, clopidogrel, aspirin combined with dipyridamole) was not recorded, and there was a significant imbalance in baseline characteristics of participants in receipt of antiplatelet therapy compared with participants who were not (table 1), and a mild imbalance in baseline characteristics in participants assigned to receive B vitamins versus placebo (table 2). The more favourable recorded effect of B vitamins in participants not in receipt of antiplatelet therapy might have been confounded by the reason they were not in receipt of the therapy—ie, B vitamins might have been more effective in patients of east Asian origin or patients with cardioembolic ischaemic stroke or intracerebral haemorrhage (who tend not be given antiplatelet drugs). However, we adjusted for the effects of this imbalance on the rates of each vascular outcome in our Cox multiple regression analysis. Through our Cox analysis we identified that, after adjusting for these effects, the use of antiplatelet therapy at baseline was a significant, independent predictor of the incidence of major vascular events (p<0·0001) and that there was a significant interaction between antiplatelet therapy and treatment with B vitamins on the primary outcome (adjusted p for interaction=0·0204), stroke (adjusted p for interaction=0·0134), and death from vascular causes (adjusted p for interaction=0·0225). We acknowledge the possibility of residual imbalance in other, unmeasured, prognostic factors at baseline, for which we could not adjust our analysis, and that such residual confounding after adjusting for imbalances in measured prognostic factors (eg, haemorrhagic stroke, cardioembolic ischaemic stroke) could affect our results. We also acknowledge that our findings might represent not an interaction of B-vitamin supplementation with antiplatelet therapy but a significant effect of lowering homocysteine by B-vitamin supplementation in patients with haemorrhagic stroke or cardioembolic ischaemic stroke.

If our findings are valid, the mechanisms by which raised homocysteine might impair vascular function in the absence of antiplatelet therapy remain to be ascertained. Laboratory investigations suggest several potential mechanisms, including impairment of endothelial function, oxidation of low-density lipids, increased monocyte adhesion to the blood vessel wall, increased lipid uptake and retention, activation of inflammatory pathways, stimulatory effects on smooth-muscle-cell proliferation, and prothrombotic tendency mediated by activation of coagulation factors and platelet dysfunction.^11^, ^12^, ^13^ If antiplatelet therapy really does modify the effects of lowering homocysteine on vascular outcomes, this might be mediated by direct effects of antiplatelet drugs on platelet activation and thrombus formation, or indirect effects of antiplatelet drugs, such as aspirin, in reducing vasoconstrictor tone, vascular smooth-muscle-cell proliferation, and release of inflammatory cytokines, oxygen radicals, and growth factors.^10^

In conclusion, our findings of a significant interaction between antiplatelet therapy and the effect of B vitamins on the primary outcome, in our exploratory analysis of an independent group of patients with previous stroke or transient ischaemic attack, support the hypothesis generated from other studies that antiplatelet therapy might modify any potential benefits of lowering homocysteine with folic-acid supplementation in the secondary prevention of major vascular events. Rather than antiplatelet therapy negating all of the effects of lowering homocysteine, it is also possible that lowering homocysteine might have a small benefit independent of antiplatelet therapy and a larger benefit in the absence of additional prophylactic antiplatelet therapy.

The external validity of our findings can be assessed more reliably by means of a meta-analysis of the relevant data from all individual patients enrolled in trials of B vitamins to prevent both stroke and ischaemic heart disease events. If validated, the implications of the findings for clinicians are that B vitamins might have a role in the prevention of vascular events in individuals at high risk but who have an allergy to, intolerance of, or lack of indication for antiplatelet therapy, such as those who are also at risk of bleeding events (eg, haemorrhagic stroke).
